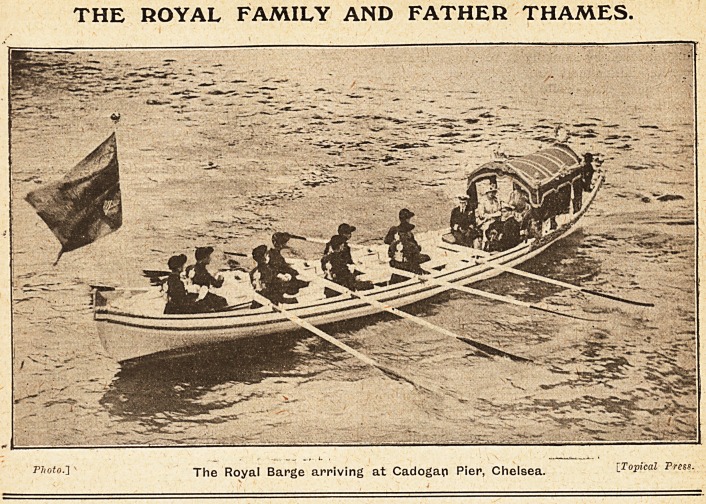# The Great Water Pageant

**Published:** 1919-08-09

**Authors:** 


### THE ROYAL FAMILY AND FATHER THAMES.

*Photo.*] The Royal Barge arriving at Cadogan Pier, Chelsea. [*Topical Press.*

### THE GREAT WATER PAGEANT.

### Tens of Thousands of People Present.

It would be impossible within the space at our command to attempt to do justice to, much less to give a detailed description of, the Water Pageant and the glorification of the Merchant Navy which, starting at London Bridge at 4 P.M. on the 4th inst., and led by the King and Queen in the King's Barge, made a triumphant progress up the Thames, passing Lambeth Bridge, to the last scene of the Pageant within the Borough of Chelsea by Cadogan Pier, which was elaborately decorated. There the King and Queen, with the members of the Royal Family, alighted and took their places in the strikingly beautiful Royal Pavilion. The naval boats passed to one side and began to fire 21 guns in salute. The Royal Barge circled up to the Bridge, turned, and made for the Pier, whilst "God Save the King" was sung by the many thousands of people assembled on both sides of the river and on the Albert and Chelsea Bridges, where there was an immense flash of handkerchiefs, and the air was filled with cheering. On landing, the King and Queen, Queen Alexandra, the Prince of Wales, Prince Albert, Princess Mary and Princess Victoria moved up to the Pier. Then the Corporation was presented and shook hands, after which a charming little girl with a strikingly beautiful bouquet presented it to Their Majesties, and the Royal Party proceeded to their position in the Royal Pavilion and arrived there just in time to receive the royal salute from the naval gunners, who, with wonderful precision, tossed oars and "'offed caps."

Accompanying the Royal party were the Lords of the Admiralty, followed by the Duke of Connaught and the Brethren of Trinity House. The Chairman of the County Council, the Mayor of Chelsea, and the Heads of the Mercantile Marine also received His Majesty. Then many boats and launches landed their distinguished occupants, until the embankment between the Pier and Pavilion, as the *Times* reports, "was a mass of white and gold hats, topped hats and cocked hats." During the review, as each class of vessels passed, there was a better opportunity to study them at closer quarters and appreciate special features. This was especially true of the barge on which was mounted (1) a small gun that was used against the Armada; (2) a model of a gun used in Nelson's day; and (3) an 18-inch gun, the heaviest piece of ordnance used afloat during the war; the sister ships of the *Mimi* and *Touton*, the vessels which were carried 3,000 miles overland to Lake Tanganyika in the East African campaign. Between the Bridge and the Pier was a special scheme of decorations, where the choir and musicians were placed at the back. In front was the Band of the 2nd Life Guards in khaki, and behind were the members of a large choir provided by the League of Arts dressed in many-coloured costumes,

**The Water Pageant on Father Thames**—(*cont.*).

of whom the *Times* records that some of its male members, "with atrocious long hair," spoilt the mediæval touch by smoking in between the chants. Decorum and manners both necessitate, and will no doubt ensure, that before these same offenders are invited to appear at any similar function, steps will be taken to teach those offending, decorum, and enforce it. For once in a way it may be said with truth that the slowly moving tugs, with civilians bare-headed and officers at the salute, were impressive and formed a striking group, though to those who did not witness the scene it may not unnaturally be thought that beauty and impressive features could not form a picture such as that actually presented on this occasion. The representatives of the sea power of the Empire went past with the halo of continuous cheering and created the keen interest of all present, the members of the Royal party showing their keenness, the King at the salute, pointing out features to his family, the Queen never resting from asking a multitude of questions, the Prince of Wales all attention, and probably the quietest and most observant member of them all. Thus the men who had made history, who went down to the sea fearless in the face of peril and made England safe, full of great deeds and splendid courage, received the most magnificent, united, and whole-hearted welcome of the Nation and Empire that Father Thames has ever exhibited in his history.

THE KING'S BARGE.

The Royal Barge excited the interest and attention of everybody. Built 230 years ago by the order of William III., it possesses the unique distinction of being the sole survivor of the wonderful and very numerous barges which in former generations added to the gaiety of the river. Ten kings and queens of England have possessed the use of it for their state progresses on the Thames, and in relatively recent years King Edward used it to attend the 4th of June celebrations at Eton in 1904. King George, too, in 1912, went in it to Henley Regatta. It is painted scarlet and white, with gold ornamentation; whilst its crew were dressed in ancient livery, scarlet, and black velvet caps. The steady sweep of their scarlet painted oars, with the Royal Standard flying from its bows, although the barge was dwarfed in appearance by the size of the river and surroundings, gave a picturesque appearance and was in bright contrast to the dull Thames water.

The best of the decorations to the bridges was undoubtedly that of Westminster, where, on the St. Thomas's Hospital side of the river, a number of boats were anchored and crowded by sightseers, which gave those who were on the House of Commons Terrace a prospect and clearness absent in some other parts of the route. St. Thomas's Hospital was crowded with patients, and its embankment and the roofs of some of its buildings were crowded with visitors, governors and friends of the institution, old patients, and some thousands of others. Like everything else which St. Thomas's undertakes, it was thoroughly well organised, and those who had the privilege of being present not only had a good view but were treated with most welcome hospitality after the procession had passed, which sent them on their homeward way in the best of spirits and content. Some portions of a hospital are scrupulously avoided, but we have the privilege of knowing that on the day of the great pageant the roof of the pathological block of St. Thomas's Hospital afforded the best possible view of the procession, by enabling those who were fortunate enough to be there, to examine in perspective the various portions of it as it came through the bridge towards Lambeth, and as it passed on its journey onwards. The hospital itself was profusely decorated. The child patients had the afternoon of their lives, for they had the place of honour in some of the big windows, and attracted the sympathy and notice of very many who were present. Ample provision was made, and full facilities given to the patients, both warrior and civilian, and the nurses, who proved devoted attendants, moved about in their really attractive uniforms with commendable quietness and generous consideration. There was a fine view of the Houses of Parliament opposite, which were bedecked with the flags of the nations of many types on a grand scale, so that the general effect was not only striking but welcome and attractive.

THE ROUTE FROM LONDON BRIDGE TO CADOGAN PIER.

Everywhere throughout the course, some five miles in length, both banks of the Thames and every building and place of vantage were crowded, usually densely crowded, with spectators. Many hundreds of thousands of people must have been present, and the crowds were so dense, sometimes nine or more deep, that those who had the privilege of passing over the route were impressed with the view that the number of people present must have been greater than has hitherto attended any of the numerous festivals, galas, and processions which have formed a continuous feature of the peace rejoicings since Peace was signed. It is a wonderful fact, affording convincing evidence of the individual interest and orderliness of the majority of the inhabitants of these islands, that throughout the proceedings in connection with the water pageant Father Thames had, we hope, the gratification of knowing that few or none suffered injury or accident, and the few casualties were of the slightest. We can only hope that London will keep up this record while she, with other great cities and communities in these islands, passes through "the whirlwind strikes and other mad and criminal agitations" which the least intelligent members of the community in various places, who have gained for themselves the name of the wild men and women, have suffered themselves to indulge in. Be this as it may, it was a grateful sight to see these vast crowds over a five, or rather a ten-mile length, when the pageant was concluded, turn away home with orderly directness and happy, satisfied faces. This was the case everywhere. The people had had the satisfaction of seeing and cheering the noble men of the sea of all ranks, from the highest to the

**The Water Pageant on Father Thames**—(*cont.*).

lowest, to whose continuous devotion, bravery and prowess we are so greatly indebted for our freedom and safety during the terrible war, which is now, we hope, rapidly drawing to its end. We can only hope that the cheering throughout of the people on shore, and of the men in the boats, may be accepted as testifying that every one who took part in the pageant or procession, as participant or spectator, will remember for all their lives with the deepest satisfaction this memorable visit to Father Thames, and much that happened to cheer and satisfy and uplift all classes of men, women, and children throughout the nation and Empire.

Father Thames.

Students of history, and all of us who have spent most of our lives as residents in the Metropolis of the Empire, must have realised on the 5th instant, when considering the scene and incidents on the Thames, how much we owe to our great river, and how inspiring and encouraging its history in fact proves to all—and they should be the overwhelming majority of British people—who make it their business to become familiar with all that has happened in the centuries that have passed. The Clarendon Press publishes "The History of England" which contains Mr. Rudyard Kipling's poem on the Thames, written by him in collaboration with Mr. G. R. L. Fletcher. The *Daily Telegraph* obtained permission to reprint this poem, and we, greatly daring, venture to reproduce it in our columns, where it will be welcomed and read by members of the medical and nursing professions, and a multitude of wounded and sick inmates of our hospitals throughout the Empire.

## Figures and Tables

**Figure f1:**